# Geriatrician telephone support for emergency medical dispatchers: safe alternatives for older patients at high risk of short-term emergency department visits

**DOI:** 10.1007/s41999-026-01483-1

**Published:** 2026-04-20

**Authors:** Xavier Dubucs, Léa Colomar, Orhiane Dabasse, Yves Rolland, Sandrine Charpentier, Sophie Elmalem, Sara Vienne-Noyes, Zara Steinmeyer, Charles Henri Houze-Cerfon, Vincent Bounes, Pierre Roucolle, Laurent Balardy, Hélène Villars

**Affiliations:** 1 Emergency Department, Place du Docteur Baylac, Toulouse University Hospital Center, 31300 Toulouse, France; 2 Centre d’Epidémiologie Et de Recherche en Santé Des Populations, INSERM 1295, Université de Toulouse, Toulouse, France; 3 Geriatric Department, Gerontopôle Toulouse University Hospital, Toulouse, France; 4 IHU HealthAge, Institute On Aging, Toulouse University Hospital, Toulouse, France; 5 Toulouse University Hospital, SAMU 31, Toulouse, France

**Keywords:** Emergency medical service communication systems, Emegerncy medical dispatcher, Older patients, Triage, Emergency department

## Abstract

**Key summary points:**

**Aim:**

For older patients at presumed high risk of short-term emergency department visits but without immediate hospital needs, we aim to describe a system that provides geriatrician telephone support to emergency medical dispatchers, offering a safe alternative to emergency department referral.

**Findings:**

Providing geriatrician telephone support to emergency medical dispatchers could offer an alternative to referring certain older patients at presumed high risk of a short-term emergency department visit. Among older patients for whom an emergency department referral was not initially recommended, some experienced early unplanned hospital visits, highlighting the need to develop and strengthen reactive alternatives to emergency department referral.

**Message:**

Collaboration between emergency medical dispatchers and on-phone geriatricians could provide a safe alternative to emergency department referrals for older patients at presumed high risk of short-term ED visits.

**Abstract:**

**Purpose:**

To describe a system enabling Emergency Medical Dispatchers (EMD) to access direct geriatrician telephone support (SCAS: Senior Care Access System) with the purpose of providing alternatives to Emergency Department (ED) referral for older patients at presumed high risk of short-term ED visits.

**Methods:**

This prospective study was conducted at the EMD of Toulouse University Hospital. EMD could contact the SCAS for patients aged 75 or older who were presumed to be at high risk of a short-term ED visit but did not require an immediate ED referral. The primary outcome was the alternative to ED referral decided by the SCAS, which included telephone advice, geriatric consultation, geriatric day hospital admission, admission to post-acute care and rehabilitation, or direct admission to an acute geriatric unit. Secondary outcomes were i) alternative follow-up destinations and ii) early unplanned hospital visits (ED visit or unplanned hospital admission within 7 days following the SCAS call).

**Results:**

A total of 364 patients were included between September 1, 2023 and February 28, 2025. The mean age of the patients was 87.5 years (± 8.3), and 40.1% were male. The primary reason for the call was altered general health status (30.8%), followed by falls (17.9%). An alternative to ED referral was proposed for 287/364 patients (78.8%). Among the 117 patients for whom the SCAS decided no ED referral or admission to an acute geriatric unit, 32 (27.4%) patients experienced an early unplanned hospital visit within a median delay of 3 days.

**Conclusion:**

Our study suggests that geriatrician telephone support for EMD could offer an alternative to ED referral for older patients with presumed high risk of short-term ED visit.

**Supplementary Information:**

The online version contains supplementary material available at https://doi.org/10.1007/s41999-026-01483-1.

## Introduction

Global population ageing combined with social changes and challenges in primary care accessibility, has contributed to the recent increase in emergency department (ED) visits among older patients [1, 2]. Currently, 22% of patients presenting to the ED are aged 65 years or older [1]. Population projections for 2050 estimate a 42% increase in this age group [2]. Approximately half of older patients' ED visits are preceded by a call to the Emergency Medical Dispatch (EMD) center [1]. The EMD will likely play a critical role in the coming years in better identifying older patients who require transfer to the ED. As demonstrated by Eastwood et al., systematic phone calls to the EMD may have the potential to reduce inappropriate ED visits [3]. However, field studies show that EMD operators most frequently decide to transfer older patients to the ED. This trend is likely explained by time constraints and the complexity of assessing older patients, who often present multiple comorbidities [4, 5].

Current evidence on the collaboration between EMD systems and geriatric care providers is limited, reflecting a significant gap in the integration of prehospital and specialized geriatric services [6, 7]. Furthermore, recent studies have proposed various models of collaboration yielding divergent results. In a previous study, we suggested that strengthening the connection between nursing home staff and the EMD could reduce the number of potential inappropriate resident transfers to the ED [8]. Another initiative involving training for EMD operators showed no effect on reducing ED visits among older patients [5]. In parallel with the initiatives implemented within EMD systems, new geriatric hotlines have recently been introduced. The purpose of these hotlines is to offer general practitioners direct phone access to a geriatrician for expert advice or assistance in patient orientation, the aim being to potentially reduce ED visits for older patients [9, 10].

Linked to the EMD, the "silver triage" system was also recently proposed in England. This initiative connects on-site paramedics in prehospital settings with a geriatrician by phone to guide decisions regarding patient transfer to the ED. A pilot study of this system showed that 80% of calls did not result in a transfer to the ED [11]. To our knowledge, no study has evaluated a system enabling EMD personnel to directly contact a geriatrician by phone to propose alternatives to ED referral for older patients. Since September 2023, such a system entitled SCAS has been implemented at the EMD of Haute-Garonne (southwestern France). The primary objective of this study was to assess the proportion of alternatives to ED referral proposed for older adults contacting the EMD, through geriatrician telephone support via the SCAS. Secondary outcomes were i) alternative follow-up destinations and ii) early unplanned hospital visits (ED visit or unplanned hospital admission within 7 days following the SCAS call) among patients who were not referred to the ED during the initial call.

## Methods

### Setting and study design

This prospective, single-centered and observational study was conducted at the EMD of Toulouse University Hospital over 18 months, from September 1, 2023, to February 28, 2025. All patients calling the EMD whose calls were transferred to the SCAS were included in the study.

Emergency Medical Services in France are coordinated by a sole dispatch center (EMD) settled in each French department and reachable by a unique national emergency number and working 24 h a day. In case of a medical emergency, EMD can be reached by both community-dwelling people or healthcare practitioners. Based on the medical situation assessment, the EMD may propose: medical advice or follow-up by phone, referral to a general practitioner or to the ED, or the dispatch of an ambulance or medical response vehicles in life-threatening situations, as appropriate. Each EMD must be composed of both medical regulatory assistants and emergency physicians. Based on a medical phone assessment, the EMD may propose medical advice or follow-up on the phone, patient referral either to general practitioner or to ED, ambulance or medical response vehicles dispatch [12].

This study was conducted at the EMD of Haute-Garonne, a department in the southwest of France with approximately 1.5 million inhabitants. The EMD is based at Toulouse University Hospital and receives about 325,000 patient calls annually.

**Fig. 1:**
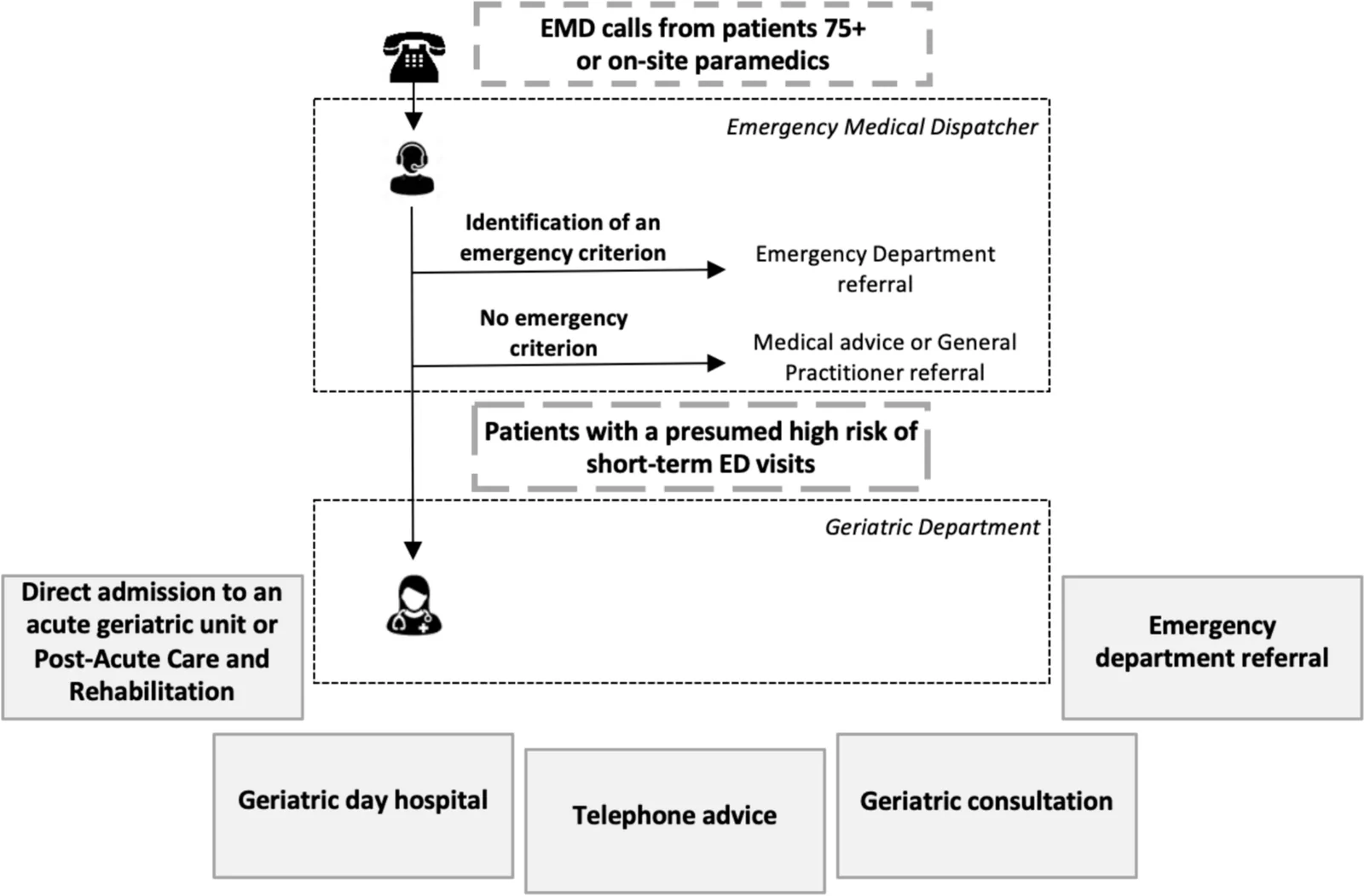
Senior Care Access System (SCAS)

Implemented on September 1st, 2023, the SCAS enabled EMD personnel to reach a geriatrician at Toulouse University Hospital during business hours (Monday to Friday, 9 AM–6 PM). Its primary objective was to offer an alternative to ED referrals for older patients who do not need such intensive medical resources. The protocol for this system is outlined in Fig. 1. This protocol (SCAS contact procedure) was presented to all EMD personnel, including medical regulatory assistants and emergency physicians, at the beginning of its implementation and was regularly reinforced through email reminders. Calls concerning patients aged 75 or older were received by the EMD. Medical Regulatory Assistants and Emergency Physicians identify the reason for the call and collect administrative data. EMD personnel could contact the SCAS if a patient was considered at high risk for a short-term ED visit but did not require immediate referral. The involvement of the SCAS was deliberately designed to be flexible to accommodate the time constraints inherent in EMD operations. Thus, the classification of "high risk for a short-term ED visit" was not defined by specific criteria but was instead estimated by EMD personnel during the call.

The EMD then explains the medical situation to the geriatrician. Depending on the situation, the geriatrician either received the call directly or called the patient back within the hour. They then propose a care plan, which may involve telephone advice, a geriatric consultation, day hospital admission, post-acute care and rehabilitation, direct admission to an acute geriatric unit, or referral to the ED. This system can also be activated when an ambulance has been dispatched on site. In such cases, paramedics transmit their assessment to the EMD, which then contacts the geriatrician according to the previously described procedure.

### Inclusion criteria

All consecutive patients calling the EMD whose calls were transferred to the SCAS were included in the study.

### Data collection and study variables

Data were prospectively collected from the patient’s medical records and compiled using the EasyMedStat platform. Demographic variables included sex, age, place of residence (home alone, home with relatives, nursing home, senior residence), and number of medications. Frailty was assessed using the Clinical Frailty Scale (Robust 1–3; Vulnerable-Frail 4–6; Severely Frail 7–9). Any indication of cognitive impairment—whether documented in the patient’s medical record or reported by the patient or relatives—was considered cognitive impairment. Comorbidities were also recorded (yes/no), including cardiovascular, pulmonary, neurological, neoplastic, and digestive-urological conditions. The reason for calling the EMD was defined as the primary concern identified during the initial contact with the patient (altered mental status, fall, acute behavioral disorder, biological abnormality, pain, infectious disease, cardio-respiratory distress, social problem, or delirium). For non-transferred patients, follow-up at day 7 was conducted by reviewing the patient’s electronic medical record to identify any early unplanned hospital visit.

### Outcome measures

The primary outcome was the alternative to ED referral decided by the SCAS, which included telephone advice, geriatric consultation, geriatric day hospital admission, admission to post-acute care and rehabilitation, or direct admission to an acute geriatric unit.

Secondary outcomes were i) alternative follow-up destination (telephone advice, geriatric consultation, geriatric day hospital admission, admission to post-acute care and rehabilitation, or direct admission to an acute geriatric unit); and ii) early unplanned hospital visits (ED visit or unplanned hospital admission) to Toulouse University Hospital within 7 days following the SCAS call.

### Statistical analysis

The prevalence of alternatives to ED referrals was described using frequencies and 95% confidence intervals. The incidence of early unplanned hospital visits was calculated for patients for whom the SCAS initially opted not to refer to the ED or admit to an acute geriatric unit (instead offering telephone advice, a geriatric day hospital visit, post-acute care and rehabilitation, or a geriatric consultation). Categorical data were reported with frequencies and percentages. Quantitative data were reported as mean with standard deviation (SD) or median with interquartile range (IQR) when the distribution was not normal. Normality was verified through the Shapiro–Wilk test, complemented by an analysis of skewness and kurtosis. No imputation was performed; the number of missing data for each variable was reported. Statistical analyses were conducted using Stata BE 19.5.

### Ethics

In accordance with French ethical law, patients (or their relatives in case of cognitive impairment) were informed that their anonymized data would be used for the study. Under French ethical and regulatory law (Code de la Santé Publique), prospective studies based on routine care data are not required to undergo ethics committee review but must be declared or covered by a reference methodology from the Commission Nationale de l’Informatique et des Libertés (CNIL). A system for the collection and computerized processing of personal and medical data was implemented to analyze the research results. Toulouse University Hospital signed a compliance commitment to the CNIL’s reference methodology MR-004. This study was approved by Toulouse University Hospital and confirms that all ethical requirements were fully respected (RnIPH 2025–76).

## Results

**Fig. 2:**
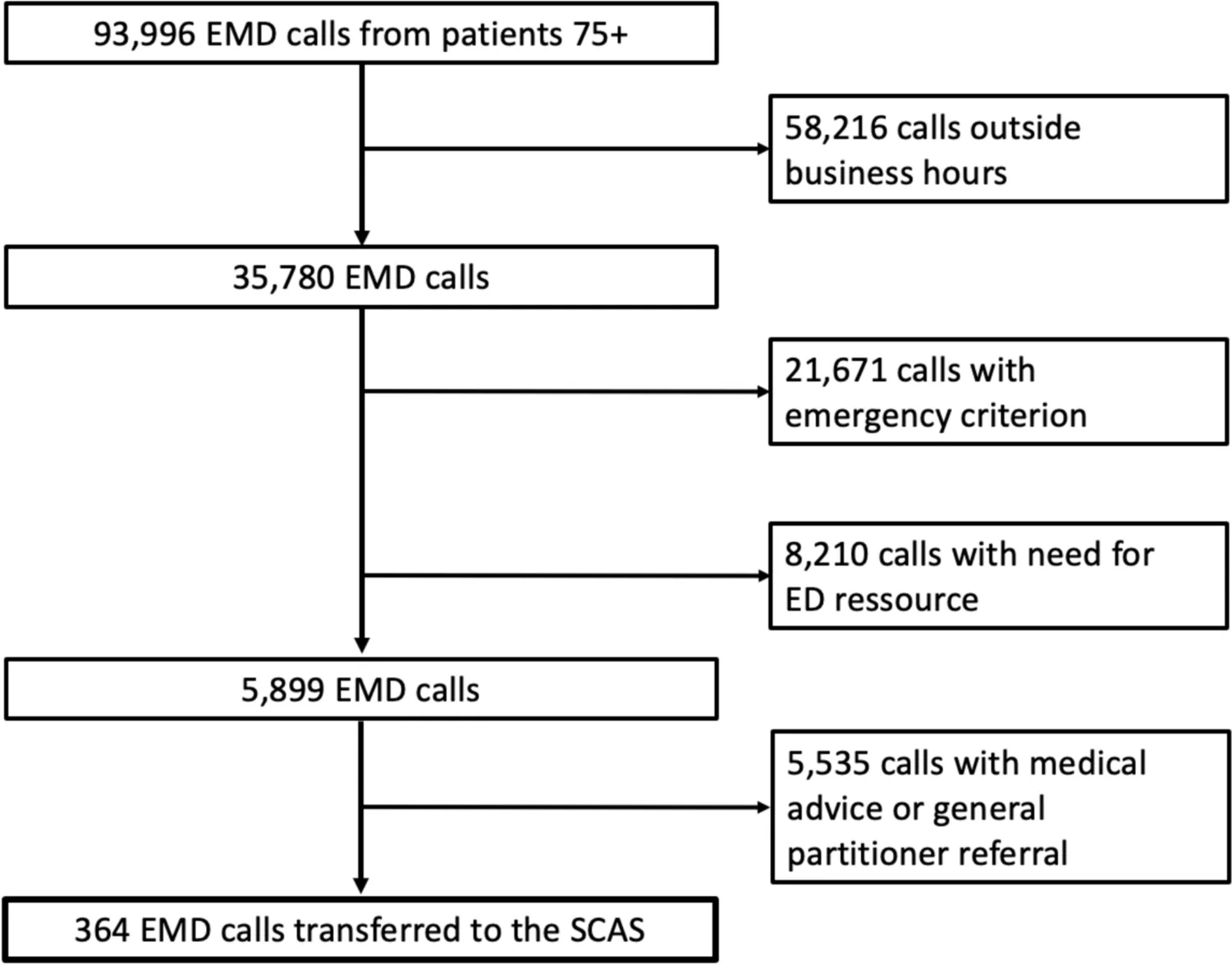
Flow-chart

During the study period, 93,996 EMD calls involved patients aged 75 or older, of which 35,780 occurred during business hours (Fig. 2). Of these, the SCAS was contacted for 364 patients, who were included in this study. The mean age of the patients was 87.5 years (± 8.3), and 40.1% were male. Patient characteristics are presented in Table 1.

**Table 1:** Patients’ characteristics

Variable	N	Mean (SD) Count (%)
Age	364	87.5 (± 8.3)
Sex, mal (%)	364	146 (40.1)
Place of residence	346	
Home, with relatives		163 (47.1)
Home, alone		118 (34.1)
Nursing Home		50 (14.5)
Senior residence		15 (4.3)
Cognitive impairment	364	200 (54.9)
Number of medications	325	6.26 (3.06)
Clinical frailty scale	320	
Robust (1–3)		87 (27.2)
Prefrail-frail (4–6)		89 (27.8)
Severely frail (7–9)		144 (45.0)
Comorbidities	348	
Cardiovascular		247 (71.0)
Digestive-urological		124 (35.6)
Neurologic		115 (33.0)
Neoplasia		63 (18.1)
Pneumological		47 (13.5)

The most frequent callers to EMD center were family doctors (44.5%), followed by patients' relatives (32.5%) and home care providers (12.5%). The primary reason for the call was altered general health status (30.8%), followed by falls (17.9%). The characteristics of these calls are detailed in Table 2.

**Table 2:** Emergency Medical Dispatcher call characteristics

Variable	N	Count (%)
EMD caller	335	
Family doctor		149 (44.5)
Relatives		109 (32.5)
Home care		42 (12.5)
Nursing Home staff		27 (8.1)
Patient himself		8 (2.4)
Reason for calling EMD	364	
Altered general health status		112 (30.8)
Fall		65 (17.9)
Acute behavioral disorder		60 (16.5)
Biological abnormality		59 (16.3)
Pain		53 (14.6)
Infectious		52 (14.3)
Cardio-respiratory distress		49 (13.5)
Social problem		44 (12.1)
Delirium		35 (9.6)

An alternative to ED referral was proposed for 287/364 patients (78.8%, 95% CI 74–83). The most common proposed decision was admission to a short-stay geriatric unit (46.7%). However, an ED referral was still decided for 21.2% of patients. Details are provided for all alternative follow-up destination and the time taken to implement these decisions in Table 3.

**Table 3:** Senior Care Access System decision

	N = 364 (%)	Median delay (Q1–Q3), days
Alternative to emergency department referral	**287 (78.8)**	
Admission to an acute geriatric unit	170 (46.7)	1 (0–1)
Telephone advice	71 (19.5)	-
Geriatric day hospital	26 (7.2)	11 (4–28)
Admission to a post-acute care and rehabilitation	10 (2.7)	1 (1–2)
Geriatric consultation	10 (2.7)	7 (6–21)
Emergency department referral	**77 (21.2)**	

Among the 117 patients for whom the SCAS decided no ED referral or admission to an acute geriatric unit (providing telephone advice, scheduling a geriatric day hospital visit, admitting to post-acute care and rehabilitation, or proposing a geriatric consultation), 32 (27.4%) experienced an early unplanned hospital visit within a median delay of 3 days (IQR 1–5.5). The primary reason for the early unplanned hospital visit was similar to the initial EMD call reason in 14/32 (43.8%) patients (Supplementary Table S1).

## Discussion

Our study suggests that geriatrician telephone support for EMD could offer an alternative to ED referral for older patients with presumed high risk of short-term ED visit. As a preliminary study, no control group was included, making it impossible to quantify the reduction in ED referrals for this population. However, an alternative to ED referral was proposed in nearly 80% of cases. Nearly half of the patients in this cohort were severely frail with multiple comorbidities. It is therefore reasonable to assume that a significant proportion of them would have been referred to the ED without this intervention. A statewide analysis of emergency medical service usage revealed that patients aged 85 and older had the highest rate of emergency medical service utilization, with 60.6% of their ED arrivals occurring via EMD. This indicates that, for this age group, EMD services serve as the primary pathway to the ED [13]. Indeed, the main innovation of this approach is to offer the EMD alternatives beyond the traditional binary decision of either referring patients to the ED or not.

The most frequently proposed SCAS referral was direct admission to an Acute Geriatric Unit (44.5%). Recent literature suggests that the mode of admission—direct hospitalization versus referral to the ED—may influence the care pathways of older patients. For example, Naouri et al. showed that patients admitted directly to acute geriatric units had shorter hospital stays compared to those transferred to the ED prior to admission [14]. Another recent multicenter study has suggested that older patients who remained overnight in the ED before hospitalization had an increased risk of morbidity and mortality compared to those admitted directly from the ED [15]. By facilitating direct admission, the SCAS could thus improve the care pathway for these patients.

The most frequent reason for SCAS activation was altered general health status (30.8%). This observation is particularly relevant, since several studies have shown that older patients presenting to the ED with altered general status or non-specific complaints exhibit increased morbidity and mortality compared to those with focal symptoms [16–18]. It could be hypothesized that offering alternatives to ED referral for these patients may be beneficial.

Finally, based on the 7-day follow-up, the SCAS proved to be a safe intervention, as only 32 patients in the entire cohort experienced an early unplanned hospital visit. Concurrently, this finding underscores the complexity of predicting care pathways for frail and comorbid patients. This observation should also encourage us to propose alternatives to reactive ED referrals by reducing delays in the management of these patients.

The relatively low number of calls to the SCAS, compared to the total number of calls received by the EMD during the same period, raises questions. Given that this system is new, there is likely an acclimatization period during which EMDs become accustomed to considering contact with the SCAS. Furthermore, we did not measure the additional time required to obtain geriatrician telephone support. In France, the median medical communication time is 3 min [19]. This brief duration may have limited the use of geriatrician support due to the inherent time constraints of EMD activities. Another point concerns the significant number of calls received outside business hours. More than half of the calls to the EMD occurred outside the operating hours of the SCAS system. Extending the availability of this system beyond business hours could be considered to increase the number of calls transferred to the SCAS.

Moreover, our study did not assess patient-reported outcomes. While there is a growing body of evidence evaluating patient-reported outcomes in out-of-hospital care for older patients, further research is needed to specifically examine these outcomes among older patients managed by emergency medical services who are not conveyed to the ED [20].

Our study has several limitations. As previously mentioned, this study lacked a control group, thereby limiting the measurement of the geriatrician telephone support effect on patient healthcare pathway. Furthermore, the incidence of early unplanned hospital visits may have been underestimated if patients were hospitalized in a facility other than Toulouse University Hospital Center. However, this risk is minimal, as patients who contact the EMD again after SCAS involvement are identified through the electronic medical record system and more easily directed to Toulouse University Hospital Center. Moreover, due to the short follow-up period, adverse outcomes beyond 7 days could not be excluded.

## Conclusion

Geriatrician telephone support for EMD could offer a safe alternative to ED referrals for older patients with presumed high risk of short-term ED visit. Further studies are needed to optimize the management of patients not referred to the ED.

## Supplementary Information

Below is the link to the electronic supplementary material.

Supplementary file1 (DOCX 21 KB)

## Data Availability

De-identified individual participant data are available from the corresponding author (Xavier Dubucs) upon reasonable request from qualified researchers.

## References

[1] DREES, « Page d’accueil DREES | Direction de la recherche, des études, de l’évaluation et des statistiques ». Consulté le: 3 novembre 2025. [En ligne]. Disponible sur: https://drees.solidarites-sante.gouv.fr/

[2] U. C. Bureau, « 2023 National Population Projections Tables: Main Series », Census.gov. Consulté le: 3 novembre 2025. [En ligne]. Disponible sur: https://www.census.gov/data/tables/2023/demo/popproj/2023-summary-tables.html

[3] Eastwood K, Smith K, Morgans A, Stoelwinder J (2017) Appropriateness of cases presenting in the emergency department following ambulance service secondary telephone triage: a retrospective cohort study. BMJ Open 7(10):e016845. 10.1136/bmjopen-2017-016845 29038180 PMC5652623

[4] Vincent A, Jomard N, Haesebaert J, Comte B, Gilbert T, Schott A-M (2019) Referral pathway of patients aged 75 years and older after a telephone triage by the French emergency medical communication center (SAMU). Arch Gerontol Geriatr 84:103893. 10.1016/j.archger.2019.05.018 31202586

[5] Foucaud A et al (2023) Evaluation of a training program for emergency medical service physician dispatchers to reduce emergency departments visits. J Am Geriatr Soc 71(2):484–495. 10.1111/jgs.18101 36317929

[6] Blauw GJ (2023) Could pre-hospital geriatrician triage be an useful intervention to prevent emergency hospital attendances for frail older patients? Eur Geriatr Med 14(5):983–984. 10.1007/s41999-023-00797-8 37231324

[7] Martinez L et al (2020) Impact of geriatric hotlines on health care pathways and health status in patients aged 75 years and older: protocol for a French multicenter observational study. JMIR Res Protoc 9(2):e15423. 10.2196/15423 32053116 PMC7055780

[8] Dubucs X et al (2021) Factors associated with emergency medical dispatcher request and residents’ inappropriate transfers from nursing homes to emergency department. Eur Geriatr Med. 10.1007/s41999-021-00574-5 34652784

[9] Goethals L et al (2023) Decreasing hospitalizations through geriatric hotlines: a prospective French multicenter study of people aged 75 and above. BMC Geriatr 23(1):783. 10.1186/s12877-023-04495-9 38017388 PMC10685561

[10] Chomette B et al (2022) Prospective multicentered study describing the health pathway of a short-stay geriatric population hospitalised through geriatric telephonic hotline. Geriatr Psychol Neuropsychiatr Vieil 20(4):429–438. 10.1684/pnv.2022.1064 36700436

[11] Jones HT, Teranaka W, Hunter P, Gross L, Conroy S (2023) What is the impact of a pre-hospital geriatrician led telephone ‘Silver Triage’ for older people living with frailty? Eur Geriatr Med 14(5):977–981. 10.1007/s41999-023-00796-9 37219726 PMC10587299

[12] Javaudin F, Penverne Y, Montassier E (2020) Organisation of prehospital care: the French experience. Eur J Emerg Med 27(6):404–405. 10.1097/MEJ.0000000000000770 33105295

[13] Platts-Mills TF, Leacock B, Cabañas JG, Shofer FS, McLean SA (2010) Emergency medical services use by the elderly: analysis of a statewide database. Prehosp Emerg Care 14(3):329–333. 10.3109/10903127.2010.481759 20507220

[14] Naouri D, Pelletier-Fleury N, Lapidus N, Yordanov Y (2022) The effect of direct admission to acute geriatric units compared to admission after an emergency department visit on length of stay, postacute care transfers and ED return visits. BMC Geriatr 22(1):555. 10.1186/s12877-022-03241-x 35788184 PMC9254499

[15] Roussel M et al (2023) Overnight stay in the emergency department and mortality in older patients. JAMA Intern Med 183(12):1378–1385. 10.1001/jamainternmed.2023.5961 37930696 PMC10628833

[16] Van Dam CS, Peters MJL, Hoogendijk EO, Nanayakkara PWB, Muller M, Trappenburg MC (2023) Older patients with nonspecific complaints at the emergency department are at risk of adverse health outcomes. Eur J Intern Med 112:86–92. 10.1016/j.ejim.2023.03.018 37002150

[17] Wachelder JJH et al (2017) Elderly emergency patients presenting with non-specific complaints: characteristics and outcomes. PLoS ONE 12(11):e0188954. 10.1371/journal.pone.0188954 29190706 PMC5708794

[18] Erwander K, Ivarsson K, Olsson ML, Agvall B (2024) Elderly patients with non-specific complaints at the emergency department have a high risk for admission and 30-days mortality. BMC Geriatr 24(1):5. 10.1186/s12877-023-04621-7 38172691 PMC10762826

[19] Bensoussan M, Vannier M, Loeb T, Boutet J, Lapostolle F, Reuter P-G (2025) Factors affecting communication time in an emergency medical communication centers. Scand J Trauma Resusc Emerg Med 33(1):6. 10.1186/s13049-024-01315-w 39806424 PMC11727792

[20] Johnson S, Bacsu J, Abeykoon H, McIntosh T, Jeffery B, Novik N (2018) No place like home: a systematic review of home care for older adults in Canada. Can J Aging 37(4):400–419. 10.1017/S0714980818000375 30176954

