# Supplementary material for: Geriatrician telephone support for emergency medical dispatchers: safe alternatives for older patients at high risk of short-term emergency department visits
Source: Eur Geriatr Med. 2026 Apr 20;17(4):2017–24. doi: 10.1007/s41999-026-01483-1 (PMC13562128; doi:10.1007/s41999-026-01483-1)
Supplement: Supplementary file 1 [file 41999_2026_1483_MOESM1_ESM.docx]

**Supplementary Table S1**: Mean reason for early unplanned hospital visits among non-transferred patients

| **Main reason for calling EMD** | **Alternative to ED referral** | **Main reason for early unplanned hospital visit** | **Delay (days)** |
| --- | --- | --- | --- |
| Acute behavioral disorder | telephone advice | Acute behavioral disorder | 4 |
| Fall | telephone advice | Fall | 1 |
| Altered general status | telephone advice | Altered general status | 1 |
| Altered general status | telephone advice | Altered general status | 3 |
| Biological abnormality | geriatric day hospital admission | Biological abnormality | 3 |
| Cardio-respiratory distress | geriatric day hospital admission | Acute behavioral disorder | 7 |
| Cardio-respiratory distress | geriatric day hospital admission | Cardio-respiratory distress | 2 |
| Biological abnormality | geriatric day hospital admission | Biological abnormality | 1 |
| Infectious | geriatric day hospital admission | Altered general status | 7 |
| Cardio-respiratory distress | telephone advice | Cardio-respiratory distress | 7 |
| Acute behavioral disorder | telephone advice | Delirium | 1 |
| Altered general status | telephone advice | Cardio-respiratory distress | 4 |
| Delirium | telephone advice | Social problem | 5 |
| Fall | telephone advice | Acute behavioral disorder | 4 |
| Acute behavioral disorder | telephone advice | Fall | 6 |
| Fall | telephone advice | Fall | 3 |
| Fall | telephone advice | Altered general status | 5 |
| Delirium | geriatric day hospital admission | Acute behavioral disorder | 6 |
| Delirium | telephone advice | Delirium | 2 |
| Pain | geriatric day hospital admission | Cardio-respiratory distress | 6 |
| Fall | telephone advice | Infectious | 6 |
| Altered general status | telephone advice | Infectious | 1 |
| Pain | geriatric day hospital admission | Altered general status | 1 |
| Biological abnormality | telephone advice | Infectious | 3 |
| Delirium | telephone advice | Social problem | 2 |
| Acute behavioral disorder | telephone advice | Fall | 7 |
| Acute behavioral disorder | telephone advice | Acute behavioral disorder | 1 |
| Social problem | telephone advice | Acute behavioral disorder | 2 |
| Altered general status | telephone advice | Altered general status | 1 |
| Biological abnormality | telephone advice | Biological abnormality | 1 |
| Acute behavioral disorder | geriatric consultation | Acute behavioral disorder | 1 |
| Cardio-respiratory distress | geriatric day hospital admission | Fall | 1 |
